# Comparison of biomechanical foot analyses between nine Flemish foot-experts

**DOI:** 10.1186/1757-1146-7-S1-A45

**Published:** 2014-04-08

**Authors:** Ingrid Knippels, Tom Saey, Inge Van den Herrewegen, Mario Broeckx, Kris Cuppens, Louis Peeraer

**Affiliations:** 1 MOBILAB, Thomas More Kempen, Geel, Belgium; 2 Faculty of Kinesiology and Rehabilitation Sciences, KU Leuven, Leuven, Belgium

## Introduction

Treatment or prevention of specific foot problems often requires an analysis of the biomechanics of the foot. These analyses can be performed by different experts. Specifically, in Flanders, they may be performed by medical doctors in orthopaedics and rehabilitation, orthopaedic technologists, or podiatrists. It is well known that there is no standardization yet of clinical methods to analyse foot biomechanics [1,2]. The purpose of this study was to investigate to what extent foot experts differ in biomechanical foot analyses. The presented data is a pilot study on 6 subjects, analysed by 9 experts. The complete study will be performed on 78 subjects by 10 experts. In that larger study, all subjects will also be analysed with advanced gait analyses methods. This to correlate the clinical data to objective, quantitative data, and develop foot typology.

## Methods

Nine Flemish foot experts; 3 podiatrists, 5 orthopaedic technologists and 1 foot surgeon performed a biomechanical analysis of the left foot of 6 adult subjects. All subjects were healthy, wearing normal shoes. There were 3 male and 3 female subjects, average age 37 (range 26 – 54). The tools used were different for all experts; ranging from podoscopes to goniometers, an instrumented treadmill and pressure plates. All experts used the techniques they normally use in clinical practice and took between 5 and 25 minutes per subject. The results of the analyses were filled in on a specially developed form, containing multiple choice questions on 13 mobility, 16 static and 18 dynamic features of the feet. Also, 10 questions on pressure related parameters were added. All experts were free to choose which questions were answered.

## Results

The results varied substantially between the 9 experts. As an example, data of 4 static parameters is summarized in Table 1. For all other parameters, agreement between experts was more or less similar, with experts disagreeing frequently.

**Table 1:** Summary of 4 static parameters for 6 subjects analysed by 9 experts. Numbers represent the number of experts that chose that option. The bold values indicate contradictory responses.

	Calcaneus in relaxed stance			Forefoot position (relative to hindfoot)			Hallux valgus			Longitudinal arch		
	
*Subject*	*Varus*	*Valgus*	*Normal*	*.Abduct*	*.Adduct*	*Normal*	*No*	*Yes*	*Extreme*	*High*	*Low*	*Normal*
1	0	5	4	2	0	4	**6**	**3**	0	**1**	**1**	7

2	0	2	7	**1**	**1**	4	9	0	0	0	3	6

3	**1**	**3**	5	2	0	4	**8**	**1**	0	5	0	4

4	0	4	5	0	0	6	**7**	**1**	**1**	4	0	5

5	0	2	6	1	0	5	**7**	**2**	0	2	0	7

6	0	2	7	0	0	6	**8**	**1**	0	0	1	7

## Discussion

We compared all analyses between 9 experts for 6 subjects. With the total of 78 subjects we will perform statistical analyses to see which parameters are performing worst. The link with gait, dynamic 3d scanning, pressure and force plate measurements will show which parameters can be measured correctly clinically, without the need of special equipment, and which parameters cannot. With the use of machine learning techniques foot types will be defined. This foot typology will also give insight in which parameters are essential to correctly determine the foot type of an individual.
